# The Great War: II. With the Wounded at the Front

**Published:** 1918-02-23

**Authors:** 


### THE GREAT WAR.

### II.—WITH THE WOUNDED AT THE FRONT.

In a former number we followed the fortunes of a wounded soldier from the battlefield to the casualty clearing station.

We will now touch on his disposal on leaving that institution. During a battle of any magnitude the prime consideration is the clearance of the battlefield, and, with certain exceptions, as soon as a trainload fit for the journey is ready it is dispatched in order to make room for others, but in less pressing circumstances careful classification is attempted —those fit for duty after a day's rest are sent back to their units, those likely to be fit in fourteen days, or in exceptional cases up to twenty-one days, are retained, provided there is room, the principle being the retention of suitable cases as near the front as possible, a system which has many advantages from a Service point of view—their return to their own unit is assured in as short a time as possible, and transport and labour are saved.

### Shell-Shock.

The most notable exception is that of shell-shock without objective signs, which cannot be hurriedly diagnosed, and so is transferred to a special hospital for further observation; this ensures the detection of cases which are not genuine, and acts as a deterrent to malingerers, for that these are not quite unknown even in our immaculate Army is illustrated by the following story related to us by a medical officer. The man in question informed his messmates that it was his intention to "work his ticket" (get invalided home) by feigning "shell-shock"; he commenced by appearing on parade with his full kit, and when asked the reason explained that he was going on leave. This he continued until he was sent to hospital for observation ("nervous"), and in the ward he occupied himself in blowing paper darts, at which he was an adept, at all and sundry; he was then put in a ward by himself, and from there transferred to a special mental hospital, where he was placed on fatigues and told to cut grass in the garden. This he did, but ate all he cut, and so was eventually relegated to his family as a harmless lunatic. His success was, however, short-lived, for one of the messmates who shared his secret, attempting to follow in his footsteps, was detected, and, lamenting aloud that he was not so successful as his friend, was overheard, with results which led to his discovery at a munition factory and restoration, through disciplinary channels, to his proper sphere of activity.

### Nerves!

On our journey to the base we paid a flying visit of engrossing interest to one of the special "nervous" hospitals. The divisions were presided over by specialists in nervous diseases, and the remainder of the staff and attendants had been carefully selected for their previous association with mental establishments. The majority of cases were slight and were quite curable, and able to be returned to duty in six weeks. These were treated by graduated physical exercises, and occupations which kept the mind constantly distracted from the subject of war. When convalescence commenced, recovery was very rapid. Of the graver cases there were two main types—coarse injuries to the brain, due to concussion caused by the explosion of projectiles in the immediate vicinity of the individual, and indirect injuries to the nerve-cells caused by the constantly repeated nervous shocks of more distant explosions. In the former category there were instances of the condition of cerebral decortication, the sufferer being in the mental and physical condition of an infant, with feeble, tottering gait, supported by his nurse, and with memory and knowledge completely lost. These were restored to their normal condition by a kindergarten system of education, the memory being very gradually revived by reference to nursery days, and speech, by a system of mimicry. These methods were most effectual, and hastened restoration by many months. Notable symptoms in the second category were delayed tachycardia appearing suddenly, cold, clammy extremities, and pain in the shins. Other cases exhibited every conceivable form of ataxic gait and of clonic spasm.

### The Ticket for Blighty"!

We now return to the clearing station; there we find joy and excitement among those who have got their ticket for the base, and here and there as you stroll through the wards you catch references to "Blinking Blighty" which indicate the hope which many of them cherish of getting beyond the base, to the home for which they long.

A policy of grouping clearing stations on railways at, or as near as possible to, stations has for some time past been adopted on the Western front; this has the enormous advantage of saving a journey in motor ambulance cars, with the consequent inconvenience to the wounded of two extra loadings and unloadings, and of economising transport, petrol, and bearers.

### Ambulance Trains.

A certain number of ambulance trains are allotted to each army: they are controlled by the Director of Railway Transport in conjunction with the officials of the French railways, and are at the disposal of the Director of Medical Services. The number of trains based on each army railhead varies from three to six, according to the degree of activity on the front concerned. At the office of each Director of Medical Services a blackboard is kept which records continuously the numbers of the various classes of wounded, sitting or lying, which are awaiting evacuation, and as soon as a load has accumulated, a train is demanded by tele-

☛ The previous article appeared on February 9, page 391.

THE GREAT WAR—(*continued*).

phone and its route indicated: during active operations one group of clearing stations will monopolise the whole train, but in ordinary circumstances contingents will be picked up *en route*. Ambulance trains are hospitals on wheels: from the earliest days of the war the ambulance train has continuously improved—from improvised cattle-trucks, third-class, second-class, first-class compartments, up to the latest luxurious type which was recently exhibited to the public in this country, before its dispatch to the Continent. All those now in use in France are exceptionally well equipped and staffed. Many of them were placed most beneficently at our disposal by the French Government and were reconstructed in France: in the common type there are wards with central passages between three tiers of stretcher beds on either side, an operating theatre, and offices; a special feature among which is the kitchen, in which it is difficult to realise that you are on a train. The staff includes several nursing sisters, the evidence of whose presence is impressed upon us by the smart and dainty appearance of the wards.

The train travels at a slow pace—about twelve miles an hour—in order to avoid any preventable discomfort to the passengers, and in consequence it has to shunt to sidings frequently to allow rapid traffic to pass: the journey, however, is not long, it is tens of miles compared with hundreds of miles in Mesopotamia and other fronts.

### Hospital Barges.

Life on the extensive canal system of Northern France is tranquil, soothing, and monotonous, conditions eminently suited to the seriously wounded, which are taken advantage of for their transport from clearing stations to special hospitals on the lines of communication, or to the base. The ordinary canal barge, approximately 120 feet by 18 feet by 10 feet high, is converted into a luxurious hospital ward of thirty beds, and used for the conveyance of head, chest, abdominal, and compound thigh fracture cases. The patients are admitted to the ward by a lift from the deck to the floor; the lift platform is fitted with legs and alternatively becomes the operating table.

At one end of the barge there is a dynamo for generating electric light, a kitchen, and quarters for the nursing orderlies and crew, and at the other end cabins for the medical officer, two nursing sisters, dispensary and stores. They work in pairs and are navigated under the direction of the "Inland Water Transport," one tug being allotted to each pair.

